# *Culicoides* (Diptera: Ceratopogonidae) in Extra-Amazonian Oropouche Outbreak Areas of Minas Gerais, Brazil: Ecological Insights into Virus Transmission

**DOI:** 10.3390/v18030361

**Published:** 2026-03-16

**Authors:** Gabriele Barbosa Penha, Elvira D’Bastiani, Mateus Ferreira Santos Silva, Maria Eduarda da Silva Almeida, Pedro Augusto Almeida-Souza, Laura W. Alexander, Danielle Costa Capistrano Chaves, Roseli Gomes de Andrade, Elis Paula de Almeida Batista, Natália Rocha Guimarães, Talita Émile Ribeiro Adelino, Luiz Marcelo Ribeiro Tomé, Bergmann Morais Ribeiro, Luiz Carlos Júnior Alcântara, Maria da Conceição Bandeira, Fabrício Souza Campos, Ana I. Bento, Álvaro Eduardo Eiras, Filipe Vieira Santos de Abreu

**Affiliations:** 1 Laboratório de Comportamento de Insetos, Instituto Federal do Norte de Minas Gerais, Salinas 39560-000, MG, Brazil; gbpenha1@gmail.com (G.B.P.); mfss5@aluno.ifnmg.edu.br (M.F.S.S.); mariaeduarda86615@gmail.com (M.E.d.S.A.); pedro.aasouza2020@gmail.com (P.A.A.-S.); 2 Programa de Pós-Graduação em Parasitologia, Instituto de Ciências Biológicas, Universidade Federal de Minas Gerais, Belo Horizonte 31270-901, MG, Brazil; 3 Department of Public & Ecosystem Health, College of Veterinary Medicine, Cornell University, Ithaca, NY 14850, USA; elviradbastiani@gmail.com (E.D.); lwa7@cornell.edu (L.W.A.); camposvet@gmail.com (F.S.C.); 4 Secretaria de Saúde do Estado de Minas Gerais, Coordenação Estadual de Vigilância de Arboviroses e Controle Vetorial, Belo Horizonte 31630-901, MG, Brazil; danielle.capistrano@saude.mg.gov.br (D.C.C.C.); roseli.andrade@saude.mg.gov.br (R.G.d.A.); elis.batista@saude.mg.gov.br (E.P.d.A.B.); 5 Setor de Arbovirologia, Fundação Ezequiel Dias, Belo Horizonte 30510-010, MG, Brazil; natyroguiman@yahoo.com.br (N.R.G.); talita.adelino@funed.mg.gov.br (T.É.R.A.); 6 Instituto de Ciências Biomédicas, Universidade Federal do Rio de Janeiro, Rio de Janeiro 21941-630, RJ, Brazil; rcelofsa_rt@hotmail.com; 7 Baculovirus Laboratory, Department of Cell Biology, Institute of Biological Sciences, University of Brasilia, Brasília 70910-900, DF, Brazil; bergmann.ribeiro@gmail.com; 8 Instituto René Rachou, Fiocruz Minas, Belo Horizonte 30190-009, MG, Brazil; alcantaraluiz42@gmail.com; 9 Laboratório de Entomologia e Vetores, Universidade Federal do Maranhão, São Luís 65085-580, MA, Brazil; mariza_bandeira@hotmail.com; 10 Laboratório de Bioinformática & Biotecnologia, Instituto de Ciências Básicas da Saúde, Universidade Federal do Rio Grande do Sul, Porto Alegre 90035-003, RS, Brazil; 11 Laboratório de Insetos Transmissores de Hematozoários, Instituto Oswaldo Cruz, Fundação Oswaldo Cruz, Rio de Janeiro 21040-900, RJ, Brazil; 12 Centro Colaborador de Entomologia/Lacoi/IFNMG/Secretaria Municipal de Saúde de Salinas, Salinas 39560-000, MG, Brazil

**Keywords:** arbovirus, *Culicoides paraensis*, midge, entomological surveillance, Oropouche, vectors

## Abstract

Oropouche fever (OF), caused by Oropouche virus (OROV), has expanded beyond its Amazonian range into Minas Gerais (MG), Brazil, raising concern about transmission in extra-Amazonian Atlantic Forest landscapes. Critical gaps persist regarding *Culicoides* vector communities, anthropophily, and climate-sensitive transmission risk in these newly affected regions. We conducted targeted entomological surveys outbreak-driven by human OF cases, standardized across five MG communities using CDC light traps and Protected Human Attraction (PHA) to characterize *Culicoides* composition. Females of *Culicoides* underwent RT-qPCR for OROV (*n* = 819) and physiological assessment (*n* = 312). We developed an entomological alert framework that integrates blood-fed abundance, minimum infection rate (MIR) upper confidence bounds, and environmental drivers (i.e., mean temperature, relative humidity and precipitation) via generalized additive mixed models, which explained 68% of the variability in *Culicoides* abundance and the alert index across communities. We collected 1171 *Culicoides* individuals representing five species (*C. leopoldoi*, *C. paraensis*, *C. pusillus*, *C. foxi*, and *C. limai*). *C. leopoldoi* (79.1%) and *C. paraensis* (20.3%) were the predominant species; notably, *C. paraensis* is recognized as the primary vector of OROV in the Americas. *C. paraensis* was documented for the first time in all five outbreak areas and dominated PHA captures (90%), suggesting anthropophily. Although no specimens tested OROV-positive (consistent with expected field infection rates of 0.01–1%), MIR upper bounds reached 132/1000 in low-sample settings and humidity and temperature strongly modulated abundance. This operational baseline and alert index transform virologically negative, sparse surveillance data into prioritized targets for intensified sampling and vector control during early, low-prevalence phases, when containment of OROV’s extra-Amazonian spread is still achievable.

## 1. Introduction

Oropouche virus (OROV) has recently emerged as a significant public health concern in the Americas, expanding beyond its historical Amazonian range into new regions and affecting previously non-endemic populations [1,2,3]. In Brazil, this expansion includes the Northeast, Central-West, Southeast, and Southern regions, with increasing reports of autochthonous Oropouche fever (OF) cases and outbreaks in densely populated areas [4]. The expansion of OROV beyond the Amazon has been linked to broader eco-epidemiological pressures at the human–wildlife interface, including environmental change, landscape fragmentation, urbanization, and increased contact among vectors, wildlife reservoirs, and human populations [5,6]. These eco-epidemiological pressures create conditions that favor viral adaptation by increasing contact among vectors, reservoir hosts, and susceptible human populations, exposing viral populations to novel selective pressures in non-endemic environments, and facilitating genomic reassortment among co-circulating OROV lineages, as recently documented [1,2]. Brazilian OF cases coincide with the first Caribbean outbreak in decades in Cuba, as well as recent outbreaks in Peru, suggesting enhanced transmission capacity [3,7].

Critically, ecological changes, including vector range expansion, shifts in community composition, or increased anthropophilic behavior, often precede detectable viral circulation by months to years, functioning as sensitive early-warning indicators of emerging transmission risk. Establishing baseline entomological data during the initial phases of geographic expansion is therefore essential for: (i) identifying primary vector species present in affected areas; (ii) quantifying vector-human contact rates; (iii) detecting viral circulation through molecular surveillance; and (iv) guiding evidence-based vector control interventions [8,9].

Oropouche fever is an acute febrile disease caused by OROV, first isolated in 1955 [10,11,12]. Over 29,000 confirmed cases occurred across the Americas since 2024 [3,4]. OROV transmission occurs through two epidemiologically and potentially overlapping cycles: an urban cycle, in which humans serve as the primary amplification hosts and *C. paraensis* acts as the principal vector, and a sylvatic cycle involving non-human primates, sloths, and other arboreal mammals, maintained by ecologically and epidemiologically undercharacterized *Culicoides* species (specifically regarding vector competence) [13,14,15,16,17]. Biting midges of the genus *Culicoides* Latreille, 1809 (Diptera: Ceratopogonidae) are small hematophagous insects widely distributed across tropical and temperate regions, occupying natural, rural, peri-urban, and increasingly anthropized environments [8,16,17,18]. Females of many species require blood meals for oogenesis and feed on a broad range of vertebrate hosts, placing several *Culicoides* species among the most important vectors of pathogens of medical and veterinary relevance worldwide [15,19]. In addition to OROV, *Culicoides* species are responsible for the transmission of economically and epidemiologically significant viruses such as Bluetongue virus and Epizootic Hemorrhagic Disease virus, whose emergence has been strongly linked to environmental change and vector range expansion [20,21,22].

Despite the growing burden of OF outside the Amazon region, *Culicoides* fauna and vector ecology remain critically under-characterized across large areas of Brazil, particularly in regions that were historically considered non-endemic [16,20]. In southeastern Brazil, the state of Minas Gerais (MG) has reported its first autochthonous OF cases only recently, with notifications beginning in January 2024, totaling 1625 OF cases through June 2025 (Figure S1), particularly in the health regions of Cataguases, Ubá, and Teófilo Otoni [4,23]. Notably, these transmission events are occurring within Atlantic Forest landscapes, which differ markedly from Amazonian ecosystems in terms of climate, fragmentation, biodiversity, and human land use [24,25,26]. This ecological shift raises critical questions regarding which *Culicoides* species are sustaining transmission, how vector–host interactions are structured in these environments, and whether local ecological conditions may favor the establishment of new transmission cycles.

We conducted targeted entomological surveys in MG municipalities with confirmed autochthonous cases to characterize *Culicoides* composition/abundance, quantify host contact via physiological status, estimate entomological transmission potential using minimum infection rate (MIR) metrics and an exploratory entomological alert index, detect OROV via RT-qPCR, and examine environmental drivers of abundance, thereby introducing a transferable framework for uncertainty-weighted vector surveillance in low-prevalence settings. By integrating surveillance with ecological modeling and uncertainty-weighted entomological alert frameworks, we provide the first comprehensive characterization of *Culicoides* communities in extra-Amazonian OF outbreak zones, linking vector structure and environment to emerging transmission risk. Even in the absence of virus-positive vectors (expected in low-prevalence arbovirus ecology), these data establish baseline information for monitoring trends, detecting epidemic signals, prioritizing resources, and informing control during early expansion phases when interventions work best, and when integrating entomological alerts with routine febrile-illness surveillance is most feasible.

## 2. Materials and Methods

### 2.1. Study Area

**Figure 1:**
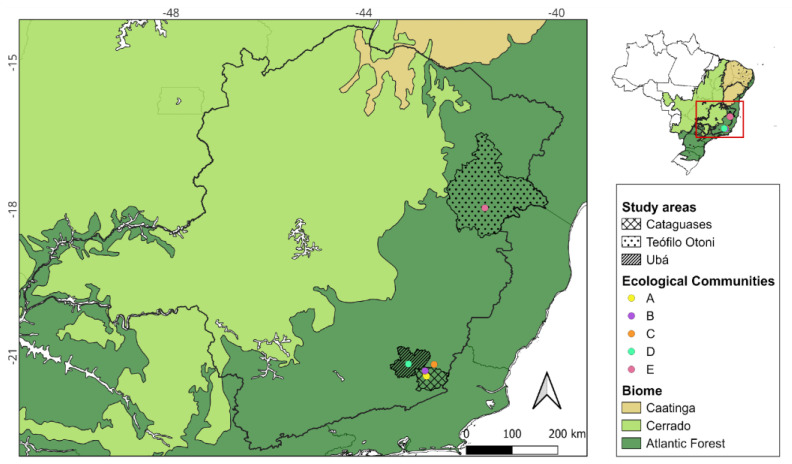
Location of the five ecological communities (A–E) distributed across three health regions and selected as study areas within the Atlantic Forest biome in the state of Minas Gerais, Brazil. These communities correspond to the spatial units used for entomological sampling and analyses.

Since early 2024, autochthonous OF cases have been confirmed in municipalities across the state of Minas Gerais, Brazil. In response, entomological collections were conducted at five sampling locations, hereafter referred to as ecological communities (A–E), based on the spatial dispersion of *Culicoides*, defined as spatial clusters with a radius of at least 5 km. These communities are located in three different health regions (Table 1, Figure 1) and were selected based on previous and recently confirmed human OF cases, representing operational ecological clusters used for entomological surveillance and analysis rather than administrative units. All communities are located in the Atlantic Forest biome (Figure 1), characterized by a tropical forest heavily impacted by anthropogenic activities, with extensive deforestation and land conversion to pastures and agricultural crops [24,25,26,27]. The climate in these regions is classified as Cwa (humid subtropical with dry winters and hot summers) for communities A–D, and Aw (tropical savanna with dry winters) for community E, respectively, according to the Köppen climate classification system, with variations in precipitation and mean annual temperature (Table 1) [27]. The geographic coordinates (latitude and longitude) of the centroid of each ecological community (A–E) are provided in Table S1 and were used for spatial analyses and mapping. The spatial distribution of these communities across health regions, along with reported human OF cases in 2025 and their location within the Atlantic Forest biome, is shown in Figure S1.

These five communities were selected for intensive sampling based on confirmed human OF clustering, accessibility for repeated sampling, presence of diverse habitat types (peridomestic, agricultural, forest edge), and representation of the primary affected health regions. This strategic sampling design prioritized outbreak-associated areas to maximize the probability of (i) detecting primary vectors; (ii) capturing vectors with recent blood meals from human hosts; and (iii) identifying virus-positive specimens if collected. By focusing surveillance efforts on locations with confirmed human transmission, this approach balances logistical feasibility with epidemiological relevance.

### 2.2. Entomological Sampling

Five ecological communities (A–E), distributed across three health regions of Minas Gerais, were sampled for entomological analyses between March and May 2025, a period that corresponds to the end of the rainy season and also peak months of reported human OF cases (Table S1, Figure S1). Within each community, sampling sites were selected according to the spatial distribution of confirmed OF cases. At each sampling site, biting midges were collected using two CDC light traps (Entomotrap, Salvador, Brazil) positioned 1.5 m above the ground, one placed in the peridomicile and the other in the extradomicile. Peridomicile environments were defined as areas immediately surrounding human dwellings that combine frequent human activity with potential *Culicoides* breeding and resting sites, including animal enclosures, shaded vegetation, and moist organic substrates. Extradomicile sites were defined as areas located farther from human dwellings, with reduced direct human activity, typically at the interface between households and adjacent vegetated or semi-natural environments. Traps were installed in sites favorable to *Culicoides* resting and breeding, such as banana plants and animal enclosures (e.g., chicken coops and pigsties). As an attractant, carbon dioxide was generated by fermenting 500 mL of sugarcane molasses in 2 L of water with approximately 7–10 g of instant dry baker’s yeast (one commercial sachet), following a fermentation-based CO_2_ generation protocol adapted [28]). The mixture was prepared in plastic containers under ambient conditions to ensure continuous CO_2_ release during trap operation [28]. Traps operated for 15 h per night (from 16:00 to 07:00) over three consecutive nights, resulting in a total sampling effort of 90 h per sampling point (15 h × 3 nights × 2 traps). Communities B, C, D, and E each had one sampling point, while community A had two sampling points. CDC light traps represent the standard approach for passive *Culicoides* surveillance globally and are particularly effective for host-seeking females in peridomestic environments. However, species-specific differences in attraction to light and CO_2_ sources can lead to collection bias, potentially underrepresenting anthropophilic species that preferentially seek human hosts through chemosensory cues beyond CO_2_ alone.

Additionally, from 15:00 to 18:00, collections were conducted using hand nets and oral aspirators through the Protected Human Attraction (PHA) method. The PHA method was therefore incorporated to complement CDC trapping and specifically target anthropophilic *Culicoides*. PHA involves trained collectors using hand-nets to capture host-seeking midges attracted to human collectors wearing protective equipment, thereby reflecting anthropophilic behavior, the most epidemiologically relevant parameter for human disease transmission. To minimize inter-individual variability in host attractiveness and ensure comparability between sites, the same trained collectors participated across all sampling locations. The PHA sampling effort corresponded to 18 h per sampling point (2 collectors × 3 h per day × 3 consecutive days. The dual-method approach employed here thus balances community-level diversity characterization (CDC traps) with targeted surveillance of the primary epidemiological threat (*C. paraensis* via PHA), addressing the limitations of single-method approaches [29].

After each trapping night and PHA session, collecting cages were labeled and transported to the field laboratory, where specimens were immobilized by refrigeration (4 °C) and sorted under a stereomicroscope. *Culicoides* specimens were then stored using two preservation methods prior to shipment to the reference laboratory: a subset (30.2%) of specimens was preserved in 70% ethanol for slide mounting, physiological condition assessment, and taxonomic identification, whereas the remaining specimens (69.8%) were cryopreserved in liquid nitrogen (−196 °C) for molecular analyses. Methods and protocols were previously approved by the Brazilian Ministry of the Environment (SISBIO nº 95162-1).

### 2.3. Physiological State and Taxonomic Identification

*Culicoides* specimens preserved in 70% ethanol were examined for physiological status under a stereomicroscope and classified into four categories: nulliparous (females that had not yet taken a blood meal); engorged (females with abdomens filled with freshly ingested blood); gravid (females with visible eggs in the abdomen); and parous (females that had previously taken a blood meal and laid eggs, as indicated by abdominal and ovarian pigmentation), according to the criteria of Dyce (1969) [30].

Following physiological classification, taxonomic identification was performed using standardized protocols for specimen clarification, slide mounting, and morphological examination, as described by Wirth and Marston [31] and complemented by the Operational Document for the Identification of *Culicoides* Latreille (Diptera: Ceratopogonidae), issued by the Pan American Health Organization [32].

### 2.4. RNA Extraction and OROV Molecular Diagnosis

*Culicoides* specimens intended for molecular analysis were removed from liquid nitrogen and immediately transferred to a cold table at −20 °C to prevent RNA degradation [24]. Specimens were preliminarily identified to the lowest possible taxonomic level based on wing morphology, following the Operational Document for the Identification of *Culicoides* Latreille (Diptera: Ceratopogonidae) [32].

Females were then grouped into pools of up to 25 individuals according to species, collection site, and engorged/non-engorged condition. Each pool was placed in a 1.5 mL microtube containing sterile beads and 300 µL of L-15 culture medium supplemented with 20% fetal bovine serum, 0.5% non-essential amino acids, 1% penicillin, 0.1% gentamicin, and 0.1% fungizone. This protocol and medium composition were selected due to their capacity to enable viral isolation, as they prevent fungal and bacterial contamination and maintain viral stability in RT-qPCR-positive OROV specimens. Samples were homogenized for 45 s at 8000 rpm using a Loccus L-Beader 24^®^ homogenizer (Loccus Biotecnologia, Cotia, Brazil), followed by centrifugation at 14,000 rpm for 8 min at 4 °C. A 140 µL aliquot of the supernatant was used for RNA extraction with the QIAamp Viral RNA Mini Kit (Qiagen, Hilden, Germany), according to the manufacturer’s instructions. RNA was eluted in 50 µL of buffer AVE and stored at −80 °C until analysis.

RT-qPCR assays for OROV detection were performed on a QuantStudio 3 Real-Time PCR System (Applied Biosystems, Foster City, CA, USA) using the GoTaq^®^ 1-Step RT-qPCR System (Promega, Madison, WI, USA) following procedures previously described by Naveca et al. (2017) [33]. Briefly, the assay targets the S-segment of OROV using the following primers and probe: OROV-F: 5′-TCCGGAGGCAGCATATGTG-3′; OROV-R: 5′-ACAACACCAGCATTGAGCACTT-3′, OROV-P: 5′-FAM-CATTTGAAGCTAGATACGG-3′-BHQ1. The thermal cycling conditions consisted of reverse transcription at 50 °C for 20 min, initial denaturation at 95 °C for 2 min, followed by 45 cycles of 95 °C for 15 s and 60 °C for 1 min.

Each RT-qPCR run included a positive control consisting of RNA from a previously confirmed OROV sample, and a no-template negative control to verify the absence of contamination. All amplification reactions were performed in duplicate, and amplification curves were analyzed using the QuantStudio Design and Analysis Software (version 2.7.0).

Molecular screening for OROV in field-collected vectors serves multiple surveillance objectives: (i) confirming that primary vectors are not only present but also potentially infected; (ii) estimating infection prevalence (minimum infection rate) to quantify transmission intensity; (iii) identifying geographic hotspots where vector infection rates are elevated; and (iv) potentially recovering viral genomic sequences for phylogenetic analysis.

### 2.5. Statistical Analysis

#### 2.5.1. Minimum Infection Rate Estimation

Estimating infection prevalence in vector populations is fundamental to arbovirus surveillance, yet poses substantial methodological challenges when infection rates are low and individual testing is resource-prohibitive. The Minimum Infection Rate (MIR) provides a conservative estimate of infection prevalence by assuming that each positive pool contains exactly one infected individual (thus representing a lower bound on true prevalence). In the present study, with zero positive pools detected, we calculated MIR estimates with 95% confidence intervals using exact Poisson methods to quantify the uncertainty associated with non-detection. The upper 95% CI of MIR, under zero positives, is calculated as −ln(0.05)/*n* × 1000 per 1000 specimens, where *n* = tested individuals. This provides the maximum plausible prevalence compatible with non-detection, enabling site comparisons without implying infection [34]. MIR does not assess competence; human OF cases confirm local transmission by unidentified vector(s). Critically, the upper confidence bound of the MIR represents a statistically plausible maximum infection rate compatible with our sampling, enabling risk assessment even in the absence of positive detections and facilitating comparison across communities with different sample sizes. MIR were calculated to estimate the frequency of OROV infection in *Culicoides* spp. The MIR was defined as the number of positive individuals divided by the total number of tested individuals, expressed per 1000 specimens. MIR calculations were performed separately for each vector species and community, as well as overall by species irrespective of community. Individual biting midges tested by RT-qPCR were grouped by species and community, and the total number tested and number of positives were determined. Exact Poisson confidence intervals (95%) were calculated for each group using the number of positive detections and the total number tested. MIR values and their corresponding lower and upper 95% confidence limits were derived from the Poisson rate estimates and multiplied by 1000 to express infection rates per 1000 individuals. Overall MIR estimates for each species were calculated using the same approach, pooling data across all communities. MIR estimates were reported alongside their 95% confidence intervals to account for uncertainty associated with low or zero positive detections.

#### 2.5.2. Uncertainty-Weighted Entomological Alert Index

We derived a community-level entomological alert index to summarize, in a single comparative metric, (i) the intensity of human–vector contact by *C. paraensis* and (ii) the statistical uncertainty surrounding OROV infection prevalence in this vector. Only females with evidence of at least one previous blood meal (engorged, gravid, or parous) were included, as these individuals represent vectors with confirmed host contact and therefore potential exposure to infection. For each community, we first standardized blood-fed *C. paraensis* abundance by dividing the total number of blood-fed females captured by PHA by the number of PHA collectors, yielding the number of blood-fed vectors per sampling unit (Equation (1)). To incorporate infection-prevalence uncertainty, this effort-standardized blood-fed abundance was multiplied by the upper 95% confidence limit of the MIR for *C. paraensis* in that community, estimated from pooled RT-qPCR results as described in Section 2.5.1. Because no pools tested positive, the MIR upper bound represents the maximum infection rate statistically compatible with our sampling effort rather than a point estimate of true prevalence. The resulting product therefore reflects an uncertainty-weighted entomological alert score, which increases when human-biting vector abundance is high and/or when the plausible range of undetected infection remains wide.

The uncertainty-weighted entomological alert index (AI) is defined as:

(1) $$Alert{\;index}_{i}=\;\left(\frac{B_{i}}{T_{i}}\right)\times {MIR}_{i}^{upper95}$$

where $B_{i}$ represents the number of blood-fed *C. paraensis* pools detected in community i, $T_{i}$ represents the number of traps (or collectors) used in that community, and ${MIR}_{i}^{upper95}$ corresponds to the upper 95% confidence limit of the minimum infection rate estimated for the species in that community. We use the upper confidence limit to provide a conservative estimate of infection risk, particularly when no positive pools are detected. To facilitate comparisons among communities, the resulting values were standardized using min–max normalization:

(2) $$Alert{\;index}_{norm,\;i}=\frac{Alert{\;index}_{i}-Alert{\;index}_{min}}{Alert{\;index}_{max}-Alert{\;index}_{min}}\;$$

This index was calculated separately for each community and then linearly rescaled to a 0–1 range using min-max normalization, where 0 corresponds to the lowest observed score and 1 to the highest, to facilitate relative comparison among communities (Equation (2)). The alert index is explicitly intended as an exploratory prioritization tool and not as a quantitative estimator of absolute transmission risk or human infection probability. This uncertainty-weighted alert index extends conventional MIR reporting by explicitly incorporating upper confidence limits into a community-level prioritization metric for early-phase arbovirus surveillance.

Because all MIR point estimates were zero, the alert index is explicitly intended as an exploratory prioritization tool and not as a quantitative estimator of absolute transmission risk or human infection probability. This uncertainty-weighted alert index extends conventional MIR reporting by explicitly incorporating upper confidence limits into a community-level prioritization metric for early-phase arbovirus surveillance.

#### 2.5.3. Standardizing Vector Abundance and Sampling Effort

Vector abundance was standardized to account for differences in sampling effort among collection events. Sampling effort was defined as the product of the number of sampling days, the number of collectors, and the number of sampling hours per day.

For each sampling event, the total number of individuals collected was calculated and paired with the total sampling effort. To correct abundance estimates for unequal effort, a generalized linear mixed model (GLMM) with a negative binomial error distribution was fitted using the number of collected individuals as the response variable. Species, community, and collection method (CDC and PHA) were included as fixed effects, and the logarithm of total sampling effort was incorporated as an offset term. This framework accounts for overdispersion while standardizing abundance to a common sampling effort. Model-based predictions on the response scale were generated to estimate effort-corrected abundance for each sampling event. To facilitate comparisons of relative vector composition within communities, predicted abundances were further standardized by dividing each event’s predicted value by the sum of predicted abundances within the corresponding community. Using the glmmTMB package in R [35], we obtained a relative-abundance metric that represents the proportional contribution of each species and sampling event to the total vector abundance within each community.

#### 2.5.4. Vector Community Diversity

Vector community diversity was quantified using the Shannon diversity index (H′), which incorporates both species richness and evenness. Effort-corrected and standardized abundance estimates were used to minimize the influence of unequal sampling effort across communities. For each community, standardized abundance values were summed by species to obtain total relative abundance per species. Missing species within a community were assigned a value of zero, indicating absence. Shannon diversity indices were calculated for each community using the vegan package in R [36], applying the Shannon entropy formula to standardized relative abundance values. An H′ value of zero indicates a community composed of a single species, while higher values reflect greater diversity resulting from higher species richness and/or more even species distributions.

#### 2.5.5. Environmental Effect on Vector Abundance

Environmental data were compiled to investigate the relationship between climatic variables and the standardized abundance of *Culicoides* across communities in Minas Gerais, Brazil, during March–May 2025, corresponding to the entomological sampling period. Weekly environmental data were used for precipitation (mean, median, minimum, maximum, and sum), air temperature at 2 m (mean, median, minimum, maximum), and air humidity (mean, median, minimum, maximum). Air humidity was represented by vapor pressure (hPa), derived from ERA5 reanalysis, which reflects atmospheric moisture availability. These climatic data were obtained from the Copernicus Climate Change Service (C3S) [37] and filtered to include four previous weeks of sampling dates.

To align temporal scales, the environmental variables were lagged up to four weeks prior to each midge sampling event. Specifically, for each sampling week, environmental data from one to four preceding weeks were matched, producing lagged predictors for each variable because these predictors can impact midge abundance. All environmental predictors were scaled to a mean of 0 and unit variance prior to modeling.

Given the high dimensionality and collinearity of environmental predictors, principal component analysis (PCA) was conducted separately for air humidity, precipitation, and temperature variables. The first principal components (PCs) from each environmental type, capturing the majority of variance, were retained to investigate the influence of environmental variables on standardized midge abundance. We implemented a generalized additive mixed model (GAMM) using the mgcv package in R. Principal components (PC) derived from precipitation (PC1_rain), air humidity (PC1_humidity), and temperature (PC1_temperature) data were used as predictors to reduce multicollinearity and summarize weekly environmental variation. Specifically, PC1_humidity loadings: relative humidity (0.62), vapor pressure hPa (0.58), precip. sum (0.42).

The model included random intercepts for both communities and species to account for repeated measures and species-specific effects. Smooth functions with a low degree of freedom (k = 5) were applied to each PC to allow potential nonlinear relationships, while avoiding overfitting, given the limited variation in the data. The model formula was specified as:

Standardized abundance ~ s(PC1_rain, k=5) + s(PC1_humidity, k=5) +s(PC1_temperature, k=5) + (1 | Community) + (1 | species)

## 3. Results

### 3.1. Entomological Results

**Figure 2:**
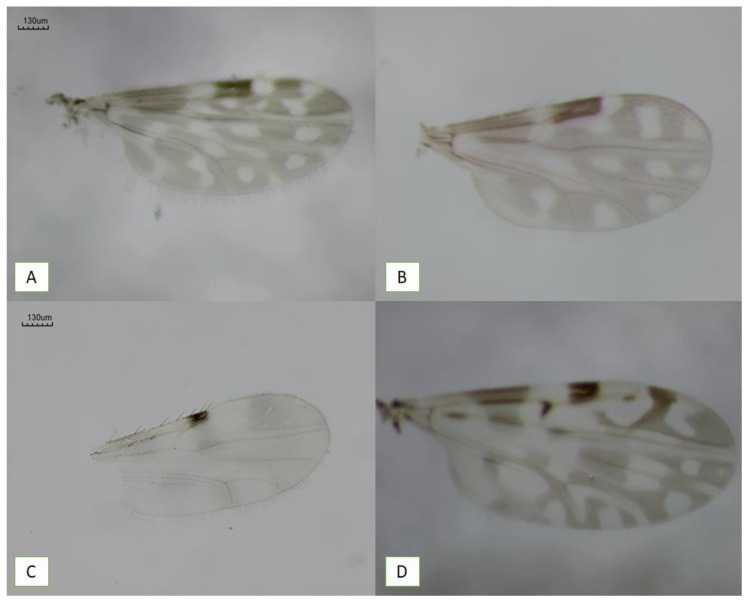
Wings of *Culicoides* species captured during entomological collections: (**A**) *C. leopoldoi*; (**B**) *C. paraensis*; (**C**) *C. pusillus*; and (**D**) *C. foxi*. Images of *C. limai* wings were not obtained due to technical limitations and the low number of specimens collected.

Across the five outbreak-associated communities sampled during the rainy season, we collected a total of 1171 *Culicoides* specimens, representing five species (Figure 2), including 1131 (96.6%) females and 40 males (3.4%). This established the first comprehensive characterization of *Culicoides* community composition in Minas Gerais areas experiencing autochthonous OROV transmission. These communities include *C. paraensis*, the primary vector implicated in urban OROV transmission throughout the Amazon basin, present in all five sampled communities, confirming the establishment of this vector in extra-Amazonian outbreak zones. The most abundant species was *C. leopoldoi*, with 926 individuals (79.08%), followed by *C. paraensis*, with 238 individuals (20.32%). The remaining species were recorded at much lower frequencies: *C. pusillus* (*n* = 5; 0.43%), *C. foxi* (*n* = 1; 0.09%), and *C. limai* (*n* = 1; 0.09%).

The highest abundance of specimens was observed in community E (*n* = 865; 73.87%), followed by community B (*n* = 215; 18.36%) and community A (*n* = 68; 5.81%), and community C (*n* = 13; 1.11%) and community D (*n* = 10; 0.85%) (Table 2).

### 3.2. OROV Detection, Physiological Status Assessment, and Capture Method Comparisons

Of the total collected specimens, 819 individuals (69.8%) were screened for OROV by RT-qPCR, organized into 42 pools. These pools included 607 *C. leopoldoi* (64.7% of all individuals of this species) and 212 *C. paraensis* (88.7%) (Table 2). The remaining specimens were preserved for taxonomic confirmation. All pools tested negative for OROV, despite the inclusion of positive and negative controls in every assay, confirming the reliability of the RT-qPCR results.

**Figure 3:**
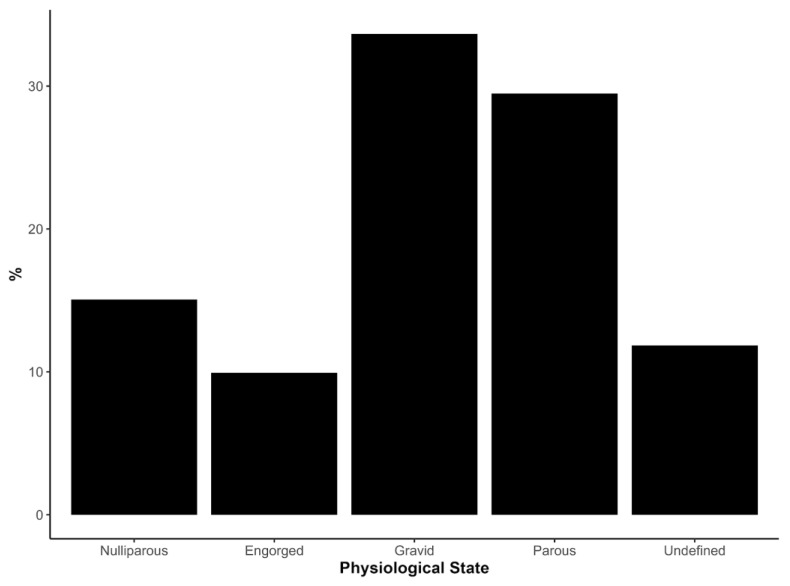
Percentage distribution of *Culicoides* females according to physiological state at the time of capture aggregated across five communities within outbreak areas in Minas Gerais, Brazil. Physiological states were classified as Nulliparous (females that have not yet completed a gonotrophic cycle, typically due to absence of a prior blood meal-aid eggs), Engorged (recently blood-fed), Gravid (carrying mature eggs), Parous (females that have previously laid eggs), and Undefined (state could not be determined). Percentages are calculated relative to the total number of specimens examined, providing a view of the reproductive status of the sampled population. Including both blood-fed and non-blood-fed individuals offers a perspective on vector biology and potential roles in transmission.

The physiological status of 312 female *Culicoides* was assessed using abdominal and ovarian pigmentation (Figure 3, Table S2). Most examined females (228; 73.1%) had already taken at least one blood meal, as evidenced by engorged, gravid, or parous classification, indicating widespread prior contact with vertebrate hosts and potential exposure to OROV (Figure 3, Table S2).

For *C. leopoldoi*, which was the most abundant species, a high proportion of females in community E were gravid (35.9%; *n* = 84), followed by parous (23.9%; *n* = 56) and engorged individuals (9.4%; *n* = 22) (Table S2). In communities A and B, gravid and parous females also predominated among the relatively smaller numbers of examined *C. leopoldoi*, and similar patterns of post-blood-meal females were observed in the C and D communities (Table S2). For *C. paraensis*, the physiological assessment was limited because most specimens from communities A, B, and E were used for molecular analysis. Nevertheless, 22 of the 26 ethanol-preserved specimens (84.6%) were classified as engorged or parous, again indicating frequent blood feeding and prior host contact (Table S2).

**Figure 4:**
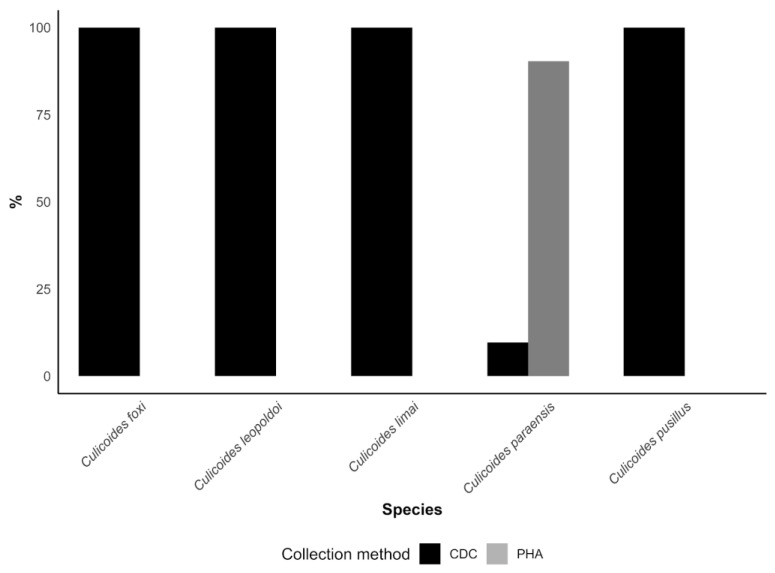
Percentage of *Culicoides* specimens captured by community vector surveillance in five communities within outbreak areas in Minas Gerais, Brazil, shown by species and collection method. Each bar represents the proportion of individuals within a species collected using either CDC light traps (black) or human landing catches (PHA, grey). Percentages are calculated for each species relative to its total count across both methods, highlighting differences in capture efficiency between CDC and PHA.

When capture methods were compared, the PHA approach proved markedly more efficient than CDC light traps for sampling *C. paraensis*, accounting for 215 individuals (90.0%) of the 239 collected. In contrast, all *C. leopoldoi* specimens were obtained exclusively with CDC light traps (Figure 4). Together, these results suggest that, despite the lack of detectable OROV RNA in tested pools, the recognized primary OROV vectors are present in outbreak areas, actively blood-feeding, and showing evidence of anthropophilic behavior, thereby fulfilling key ecological prerequisites for OROV transmission.

### 3.3. Minimum Infection Rate of OROV in Culicoides spp.

Minimum infection rate analysis was conducted on 819 *Culicoides* individuals representing two species (*C. leopoldoi* and *C. paraensis*) across 42 pools. This yielded MIR point estimates of zero for all species-community combinations, a finding that, while negative, generates informative upper confidence bounds that quantify the uncertainty associated with non-detection (Table 3). These upper confidence limits represent the maximum infection prevalence statistically compatible with our sampling effort and enable comparative risk assessment across communities despite zero positive detections.

For *C. paraensis*, the upper MIR limit was highest in community B (132.0 per 1000), followed by community A (73.8 per 1000) and community E (27.5 per 1000). For *C. leopoldoi*, upper MIR limits were lower overall, ranging from 7.9 per 1000 in community E to 26.3 per 1000 in community B. When data were pooled across communities, the upper MIR limits were 17.4 per 1000 for *C. paraensis* and 6.1 per 1000 for *C. leopoldoi* (Table 3). Smaller samples yield wider MIR confidence intervals, limiting surveillance precision.

These results indicate no evidence of active OROV infection in the tested populations. However, the upper confidence bounds suggest low but non-negligible potential OROV infection levels, consistent with the expected ecology of OROV in vector populations characterized by rare or sporadic transmission. Our uncertainty alert index ranks A (1.00: mod. BF + wide MIR), B (0.37: high uncertainty), E (0.00: high BF + narrow MIR). Prioritizes uncertainty hotspots for follow-up, not absolute risk. Stable under weight sensitivity (Supplementary Table S3).

These upper MIR bounds merit interpretation as indicators of relative uncertainty rather than infection prevalence estimates. Upper MIR bounds reflect sampling resolution: e.g., B (*n* = 28 *C. paraensis*) = 132/1000 (wide due to low *n*); E (*n* = 134) = 27.5/1000 (narrower). Inverse *n*-upper CI relation quantifies uncertainty—no evidence of infection, but sets baseline future detection thresholds. Not for threat levels absent positives/competence data. In this context, MIR provides surveillance resolution under zero-detection, aiding site comparison rather than quantifying transmission intensity or vector competence. For longitudinal surveillance purposes, repeated sampling with similar effort would enable detection of MIR increases above these baseline thresholds, functioning as a sentinel system for epidemic intensification. These MIR upper bounds provide a baseline against which future surveillance can detect increases in vector infection.

### 3.4. Uncertainty-Weighted Entomological Alert Index

Because no pools tested positive for OROV, all MIR point estimates were zero and the index reflects uncertainty-weighted entomological prioritization rather than measured differences in infection prevalence among communities. The index combines (i) the number of blood-fed *C. paraensis* captured per PHA collector (a proxy for human-vector contact) with (ii) the upper 95% confidence limit of the MIR (a measure of how high infection prevalence could plausibly be given the available data). This uncertainty-weighted alert index extends conventional MIR-based analyses by combining effort-standardized human-biting *C. paraensis* abundance with MIR upper confidence limits into a single community-level metric that prioritizes locations for early-phase arbovirus surveillance.

Raw alert scores (blood-fed *C. paraensis* per collector × MIR upper 95% CI) varied across the three communities in which both components could be calculated (A, B, and E). Community A showed the highest alert score (1291), driven by a moderate density of blood-fed *C. paraensis* (*n* = 35 across two collectors) combined with a wide MIR upper bound of 73.8 per 1000, reflecting limited sample size and therefore substantial uncertainty about true infection prevalence. Community B had an intermediate alert score (856) with fewer blood-fed vectors (*n* = 13 across two collectors) but the widest MIR upper bound (132.0 per 1000), again indicating high uncertainty. Community E, despite having the largest number of blood-fed *C. paraensis* (*n* = 44 across two collectors), exhibited the lowest alert score (606) because the MIR upper bound was narrow (27.5 per 1000), suggesting that if infection is present, it is likely at low prevalence. After min-max normalization, the alert index ranged from 1.00 (community A) to 0.00 (community E), with community B showing an intermediate value of 0.37 (Table 4). Interpreted operationally, higher index values identify communities where anthropophilic vectors are present and where infection prevalence remains weakly constrained by current sampling, highlighting locations where additional entomovirological surveillance would be most informative. The index is therefore best viewed as a comparative, uncertainty-weighted tool for prioritizing follow-up sampling rather than as a direct estimate of absolute OROV transmission risk to humans.

### 3.5. Vector Community Diversity

After accounting for sampling effort, model-based predictions revealed differences in vector abundance among species, communities, and collection methods. Shannon diversity indices varied among communities, indicating heterogeneity in vector community composition (Table 5). The highest diversity was observed in community D (H′ = 0.54), followed by community A (H′ = 0.51). Intermediate diversity levels were recorded in community B (H′ = 0.45) and community E (H′ = 0.44). In contrast, community C exhibited no detectable diversity (H′ = 0.00), consistent with the presence of a single vector species or strong dominance by a single *Culicoides* species.

Overall, these results indicate moderate variation in vector community diversity across the study area, with some communities characterized by more balanced species assemblages and others dominated by a single species. The use of effort-corrected abundance estimates ensures that observed differences in diversity reflect underlying ecological patterns rather than differences in sampling effort.

### 3.6. Environmental Effect on Culicoides Abundance

The GAMM explained a moderate proportion of the variance in standardized *Culicoides* abundance (adjusted R^2^ (explained deviance) = 0.372; Supplementary Material S1). Air humidity (PC1_humidity) and temperature (PC1_temperature) showed significant associations with standardized abundance, whereas precipitation (PC1_rain) did not reach statistical significance (*p* = 0.134). Specifically, humidity was positively associated with abundance (F = 10.96, *p* = 0.001), and temperature also showed a significant effect (F = 6.51, *p* = 0.011), while precipitation showed no significant effect (F = 2.25, *p* = 0.134) (Figure S2; Table S3). Random effects for community and species accounted for unexplained variation, capturing differences among sampling sites and midge species. The GAMM explained 68% of *Culicoides* abundance variation (R^2^_adj = 0.68). PC1_humidity, representing relative humidity/precipitation gradients, was positively associated with abundance (*p* < 0.01; df = 3.2). PC1_temperature showed a weaker positive association (*p* = 0.04), while PC1_precipitation was non-significant (*p* = 0.12).

## 4. Discussion

Our study represents the first targeted entomological survey conducted in areas of MG (five communities between March and May 2025) with confirmed autochthonous OF cases, contributing to the expansion of current knowledge on *Culicoides* fauna and OROV epidemiology in extra-Amazonian regions. OROV expansion into MG is occurring against the backdrop of a continent-wide resurgence, with >29,000 confirmed cases reported across the Americas since 2024 and sustained transmission now documented from the Amazon Basin to extra-Amazonian Brazil and the Caribbean [3,4,7]. Autochthonous outbreaks in Northeastern, Central-Western, Southeastern and Southern Brazil, together with recent epidemics in Peru and Cuba, indicate that OROV has shifted from a historically Amazon-restricted virus to a multi-biome, multi-country threat. In this context, MG functions as a critical Atlantic Forest bridge between long-endemic Amazonian foci and densely populated southeastern urban corridors, where established *C. paraensis* populations could facilitate further regional dissemination. By providing the first *Culicoides* baseline for extra-Amazonian OROV foci in MG, our study situates local entomological risk within this broader continental expansion and offers a template for other newly affected regions.

Our entomological alert index was not designed to estimate true OROV transmission probability, but to rank communities by the combination of (i) observed human-biting vector abundance and (ii) how wide the statistically plausible range of infection prevalence remains given limited sampling. Because all MIR point estimates were zero, the index is driven by the upper MIR confidence bounds, which capture how much infection could be occurring without being detected, rather than how much is occurring. Communities with many blood-fed *C. paraensis* but narrow MIR intervals are interpreted as higher-information, lower-uncertainty settings, whereas communities with fewer tested biting midges and wide MIR intervals remain higher-priority from an information-gap standpoint even if observed abundance is moderate. This design explains why community A, with moderate blood-fed densities but wide MIR bounds, scored higher than community E, which had more blood-fed vectors but narrower bounds. The same structure could be applied to other low-prevalence arboviruses where virological negatives and sparse sampling currently limit the actionability of entomological data. From a surveillance perspective, this is intentional: in early-phase, low-prevalence arbovirus systems, uncertainty about infection prevalence can be as operationally important as abundance itself because it determines where additional sampling is most needed to rule out silent transmission.

Beyond documenting vector presence, we characterized community-level entomological risk heterogeneity driven by uncertainty rather than absolute *C*. *paraensis* abundance. Community A exhibited the highest normalized alert index (1.00), combining moderate blood-fed densities (*n* = 35) with a wide MIR upper bound (73.8 per 1000), whereas community E showed the lowest index (0.00) despite higher blood-fed abundance (*n* = 44) because its narrowest MIR interval (27.5 per 1000) constrains plausible infection prevalence. This pattern illustrates how, in early-phase expansion settings, statistical uncertainty surrounding infection rates can elevate prioritization even when observed vector abundance is not maximal. The inverse relationship between sample size and upper MIR bounds highlights how sparse testing amplifies informational gaps in focal arbovirus systems.

The patterns we document, *C. paraensis* populations exhibiting anthropophilic activity, low-to-intermediate *Culicoides* diversity, and strong climatic modulation of abundance, are not unique to MG but echo conditions reported from other emerging OROV hotspots in Brazil and the western Amazon, where urban and peri-urban foci have recently intensified. These parallels suggest that the framework developed here (combined CDC-PHA sampling, MIR-based baselines, and climate-linked abundance models) can be exported to other Atlantic Forest and peri-Amazonian settings that are likely to experience OROV introduction over the coming years. These MG foci lie along a potential corridor linking Amazonian and coastal Atlantic population centers, where *C. paraensis* exhibiting anthropophilic activity could contribute to extra-Amazonian spread.

Understanding *Culicoides* species diversity and relative abundance is fundamental because vector competence varies dramatically among species, even within the same genus or different populations of the same species. Our documentation of five species across communities (*C. leopoldoi* 79.1%, *C. paraensis* 20.3%, *C. pusillus* 0.4%, *C. foxi* 0.09%, *C. limai* 0.09%) establishes baseline assemblage structure against which future shifts can be measured. Critically, *C. paraensis*, the primary vector of OROV in the Amazon Basin [13,24,38], comprised over 20% of captures and dominated PHA collections (90% of *C*. *paraensis* individuals), suggesting anthropophilic behavior. This constitutes the first record in these specific outbreak areas (previously noted only in Belo Horizonte [21], absent from Laender et al.’s (2004) [39] comprehensive MG survey). However, this information should be interpreted with caution, as cryptic species belonging to the *C. paraensis* group may occur [31].

No OROV RNA was detected in 42 pools (819 specimens), consistent with typical field infection rates (0.01–1.0%) requiring thousands screened for reliable detection [14,34]. Viral RNA in infected vectors may be transiently detectable depending on time since infectious blood meal, environmental exposure conditions, and viral replication kinetics, so absence of detection in a finite sample does not exclude ongoing transmission, but reflects the focal, heterogeneous nature of arbovirus circulation in vector populations. Physiological assessment confirmed established populations with frequent host contact. Of 312 females examined, 73.1% were blood-fed (engorged, gravid, parous), including 84.6% of *C. paraensis* and high parous rates in *C. leopoldoi* (23.9% in community E), indicating repeated feeding and local breeding. Similar engorgement patterns have been reported elsewhere in Brazil, such as in Maranhão, where many engorged *Culicoides* females were collected in rural areas [39]. While *C. leopoldoi* shows peridomestic hematophagy, there is currently no evidence supporting OROV vector competence for this species [40].

Vector community diversity patterns illuminate this risk heterogeneity. Shannon indices ranged 0.00–0.54, with low-diversity assemblages showing strong dominance by one or two species versus intermediate diversity elsewhere. Low-diversity communities may amplify *C. paraensis*’ relative contribution, potentially elevating local transmission [41,42], whereas diverse assemblages may dilute the impact of competent vectors [8]. Atlantic Forest fragmentation likely favors generalist *C. paraensis* in anthropized landscapes. Environmental drivers further shape vector abundance and diversity. Humidity (F = 10.96, *p* = 0.001) and temperature (F = 6.51, *p* = 0.011) were significantly associated with standardized abundance. Humidity sustains larval habitats and adult survival [8,43], and temperature extends activity windows and may influence viral replication [22]. These predictors enable targeting high-risk periods and sites for surveillance and align with *Culicoides* optima but imply vulnerability to climate change. Projections of rising humidity/temperatures in Southeast Brazil [8] could amplify abundance, extending transmission windows, underscoring the need for dynamic risk mapping. Together, these results indicate that ecological structure and environmental drivers jointly modulate vector abundance and community composition, and that the absence of detectable OROV in vectors does not preclude epidemiological risk. This reinforces the value of integrating entomological, ecological, and quantitative approaches for early detection and risk assessment in emerging transmission areas.

Capture methods revealed distinct patterns between species. *C. leopoldoi* was collected exclusively using CDC light traps, suggesting an association with peridomestic environments related to animal breeding, as previously reported in Brazilian surveys [16,40]. In contrast, *C. paraensis* exhibited marked anthropophilic behavior, being predominantly captured through the PHA method, consistent with studies conducted in Brazil showing a strong preference of *C. paraensis* for human hosts and the limited efficiency of CDC light traps for this species [9,44]. Similar findings have also been reported outside Brazil, including in Cuba, where *C. paraensis* was detected exclusively through human landing collections, with no records from light traps [29].

One of the most relevant findings of our study was the occurrence of *C. paraensis* across all sampled communities. Unlike the survey conducted by Laender et al. (2004) [39], which relied solely on CDC traps and did not record this species in MG, our results indicate that the inclusion of the PHA method was crucial for its detection. Indeed, approximately 90% of all *C. paraensis* individuals in our study were collected using PHA. We therefore recommend the inclusion of PHA or equivalent human-attraction methods in future entomological surveys targeting OROV vectors in extra-Amazonian regions. The entomological alert index integrates blood-fed abundance (host contact proxy) with MIR bounds, prioritizing uncertainty hotspots over high-density, low-risk zones. PHA’s efficiency for *C. paraensis* mandates its routine use alongside CDC traps.

Our study has several limitations that contextualize findings and guide future work. Methodological biases inherent to collection approaches may influence species representation. CDC light traps favor light-attracted species like *C. leopoldoi* and may under-sample highly anthropophilic *C. paraensis*, which dominated PHA captures (90%). PHA directly quantifies human-vector contact; it is labor-intensive and limited to diurnal peaks (1500–1800 h), possibly missing nocturnal activity [1]. Low Shannon diversity (0.00–0.54) reflects targeted outbreak sampling and limits inference on sylvatic cycle dynamics. The lack of forest sampling precludes assessing wild reservoirs (e.g., sloths, primates) and secondary vectors, which are critical for understanding transmission cycles. Zero OROV positives yield wide MIR bounds; larger samples or individual testing could refine prevalence estimates. The inverse sample size-upper MIR relation (small *n* → wide CIs) highlights surveillance gaps in low-prevalence systems. MIR upper bounds are sensitivity metrics, not action triggers or competence proxies—require positives/longitudinal data for intensity [34]. The absence of blood-meal identification restricts detailed inference of host preferences (e.g., microbiome and interaction with other flying blood-sucking insects), which future studies could address through molecular blood-meal analysis, metagenomics, longitudinal monitoring, and multi-habitat sampling designs. The alert index itself also has important limitations and should be interpreted cautiously. It rests on MIR upper confidence limits derived from zero-positive pools, so it cannot distinguish between truly uninfected and very low-prevalence vector populations, and it ignores several determinants of human risk (e.g., human immunity, health-care access, fine-scale biting heterogeneity), making it unsuitable for forecasting case incidence or setting absolute risk thresholds. Its main value is pragmatic: providing a transparent, reproducible way to rank outbreak-affected communities by where anthropophilic vectors are present and where infection prevalence remains most weakly constrained, thereby guiding where to intensify future entomo-virological sampling.

Our uncertainty-weighted entomological alert index highlights moderate-abundance communities, such as community B with the highest normalized score, as locations where additional entomological and virological sampling would be most informative and where targeted vector management could be prioritized. As surveillance efforts scale, larger sample sizes will narrow MIR confidence intervals, transitioning the Alert Index from uncertainty-driven prioritization (appropriate for early, low-prevalence phases) to data-driven assessments of infection prevalence. This framework particularly highlights medium-abundance communities like B, which offer the highest informational return on investment under resource constraints by balancing vector-host contact potential with unresolved statistical uncertainty. Environmental gradients associated with higher abundance (PC1_humidity, *p* < 0.01) may inform targeted surveillance intensification rather than fixed control triggers. Humidity gradients inform surveillance timing, not control triggers, as it requires validation through extended monitoring. Defining operational thresholds and lag structures requires longitudinal, high-frequency sampling beyond this study’s cross-sectional design. Routine deployment of PHA methods will enhance detection of anthropophilic species like *C. paraensis* and integrating these entomological baselines and alert metrics into national surveillance platforms [4] could support structured risk-informed surveillance mapping within existing entomological monitoring workflows and help prevent urban amplification, as observed in Northeast Brazil outbreaks [3,5]. Entomological alerts could be integrated with routine febrile illness reporting to trigger targeted field investigations when human case numbers are still low. Embedding MG within the wider extra-Amazonian and Pan-American OROV emergence underscores that entomological baselines and early-phase uncertainty metrics are not only locally useful but essential to regional efforts to prevent OROV from following the explosive expansion trajectories seen for dengue, Zika, and chikungunya across the continent. Translation to national platforms would necessitate infrastructure development, including API integration with SINAN/SIVEP, standardized entomological reporting protocols, and cross-sectoral coordination between health/veterinary/environmental agencies. Our uncertainty-weighted alert index indicates that communities A and B should be prioritized for intensified entomological and virological sampling, even in the absence of OROV-positive pools. Integrating PHA into routine *Culicoides* surveillance will improve detection of anthropophilic vectors like *C. paraensis* that are poorly sampled by CDC light traps. Vector control and community engagement efforts can be strategically timed to weeks with higher predicted humidity and temperature, when *Culicoides* abundance and human-vector contact are most likely to peak.

In conclusion, this baseline study documents the establishment of *C. paraensis* in OROV outbreak areas of MG and provides evidence of human-biting activity. By integrating diversity patterns, physiological structure, environmental drivers, and statistical uncertainty, our framework captures multidimensional entomological variation relevant to potential transmission across OF outbreak communities, establishing a scalable baseline for OROV surveillance outside the Amazon basin. Transforming sparse and virologically negative data into uncertainty-weighted prioritization tools is essential for early containment of OROV’s extra-Amazonian spread.

## Figures and Tables

**Table 1:** Climatic characteristics and reported human Oropouche fever (OF) cases in 2024 in communities across different health regions of Minas Gerais, Brazil. Ecological communities are coded according to their spatial groupings used in our study (A–E). Average temperature (°C), annual precipitation (mm), and annual average air humidity are presented as ranges or as means (minimum–maximum). Temperature and precipitation, air humidity data were obtained from long-term averages reported by Copernicus Climate Change Service (C3S).

Communities	Health Region	OF Cases	Weekly Average		
			Temperature (°C)	Precipitation (mm/day)	Air Humidity (hPa)
A–B	Cataguases	536	22.8 (18.5–30.7)	3.53 (0–82.8)	20.3 (12.7–26.8)
C–D	Ubá	341	21.4 (17.4–29.57)	3.48 (0–113)	19.1 (12.1–25.8)
E	Teófilo Otoni	77	23.2 (19.4–30.5)	2.99 (0–79.9)	20.0 (12.9–26.7)

**Table 2:** Number of *Culicoides* specimens collected and tested for Oropouche virus (OROV) in five communities within outbreak areas in Minas Gerais, Brazil. Values are presented as No./Tested, where No. indicates the total number of specimens collected and Tested indicates the number of specimens processed for laboratory detection of OROV by RT-qPCR. Species rows show the distribution of collections and testing across Communities A-E, while the Total row sums count for each community.

Species	Communities					
	A (No./Tested)	B (No./Tested)	C (No./Tested)	D (No./Tested)	E (No./Tested)	Total (No./Tested)
*Culicoides foxi*	0/0	0/0	0/0	0/0	1/0	1/0
*C. leopoldoi*	5/0	184/140	0/0	8/0	729/467	926/607
*C. limai*	1/0	0/0	0/0	0/0	0/0	1/0
*C. paraensis*	59/50	29/28	13/0	2/0	135/134	238/212
*C. pusillus*	3/0	2/0	0/0	0/0	0/0	5/0
Total	68/50	215/168	13/0	10/0	865/601	1171/819

**Table 3:** Minimum Infection Rate (MIR) estimates and 95% confidence intervals for Oropouche virus (OROV) in *Culicoides* species across three tested communities within outbreak areas in Minas Gerais, Brazil.

Species	Community	No. Tested	Positives	MIR (per 1000)	Lower 95% CI	Upper 95% CI *
*C. leopoldoi*	B	140	0	0.0	0.0	26.3
*C. leopoldoi*	E	467	0	0.0	0.0	7.9
*C. paraensis*	A	50	0	0.0	0.0	73.8
*C. paraensis*	B	28	0	0.0	0.0	132.0
*C. paraensis*	E	134	0	0.0	0.0	27.5
*C. leopoldoi*	Overall	607	0	0.0	0.0	6.1
*C. paraensis*	Overall	212	0	0.0	0.0	17.4

* Note: Upper 95% CI reflects maximum plausible prevalence.

**Table 4:** Composite uncertainty-weighted entomological alert index for Oropouche virus (OROV) across the three tested communities within outbreak areas in Minas Gerais, Brazil.

Community	Blood-Fed Vectors *(n*)	No. of PHA Collectors	Upper 95% MIR (per 1000)	Alert Index	Normalized Risk Index
A	35	2	73.8	1291	1.00
B	13	2	132.0	856	0.37
E	44	2	27.5	606	0.00

Notes: The uncertainty-weighted entomological alert index was calculated as the product of the number of blood-fed *C. paraensis* per sampling unit (PHA collectors) and the upper 95% confidence limit of the MIR. The normalized entomological alert index was obtained using min-max scaling and is intended solely for relative comparison among communities.

**Table 5:** Shannon diversity index (H′) of *Culicoides* communities across five communities within outbreak areas in Minas Gerais, Brazil.

Community	*Culicoides leopoldoi*	*Culicoides limai*	*Culicoides paraensis*	*Culicoides pusillus*	*Culicoides foxi*	Shannon Diversity Index (H′)
A	0.071	0.014	0.869	0.043	0	0.508
B	0.855	0	0.135	0.009	0	0.447
C	0	0	1	0	0	0.000
D	0.766	0	0.233	0	0	0.544
E	0.843	0	0.155	0	0.001	0.441

Notes: Shannon diversity indices (H′) were calculated using effort-corrected and standardized abundance estimates derived from model-based predictions. An H′ value of zero indicates a community dominated by a single species.

## Data Availability

The data and code used in our study are available at: https://github.com/elviradbastiani/OROV_Minas_Gerais_2026 (accessed on 10 February 2026).

## Supplementary Materials

The following supporting information can be downloaded at: https://www.mdpi.com/article/10.3390/v18030361/s1, **Figure S1.** Distribution of *Orthobunyavirus oropouchense* (OROV) cases in Minas Gerais, Brazil. **Figure S2.** Partial effects of climatic principal components on standardized abundance estimated using a generalized additive mixed model (GAMM). **Table S1.** Geographic coordinates and characteristics of the ecological communities (A–E) sampled in Minas Gerais, Brazil. **Table S2.** Physiological state of female *Culicoides* captured in five communities within outbreak areas in Minas Gerais, Brazil. **Table S3.** Results of a Generalized Additive Mixed Model (GAMM) assessing the relationship between standardized Culicoides abundance and three principal components summarizing climatic variation. **Supplementary Material S1.** GAMM output.
