# Supplementary material for: Culicoides (Diptera: Ceratopogonidae) in Extra-Amazonian Oropouche Outbreak Areas of Minas Gerais, Brazil: Ecological Insights into Virus Transmission
Source: Viruses. 2026 Mar 16;18(3):361. doi: 10.3390/v18030361 (PMC13030872; doi:10.3390/v18030361)
Supplement: Supplementary file 1 [file viruses-18-00361-s001.zip › viruses-4180097-supplementary.pdf]

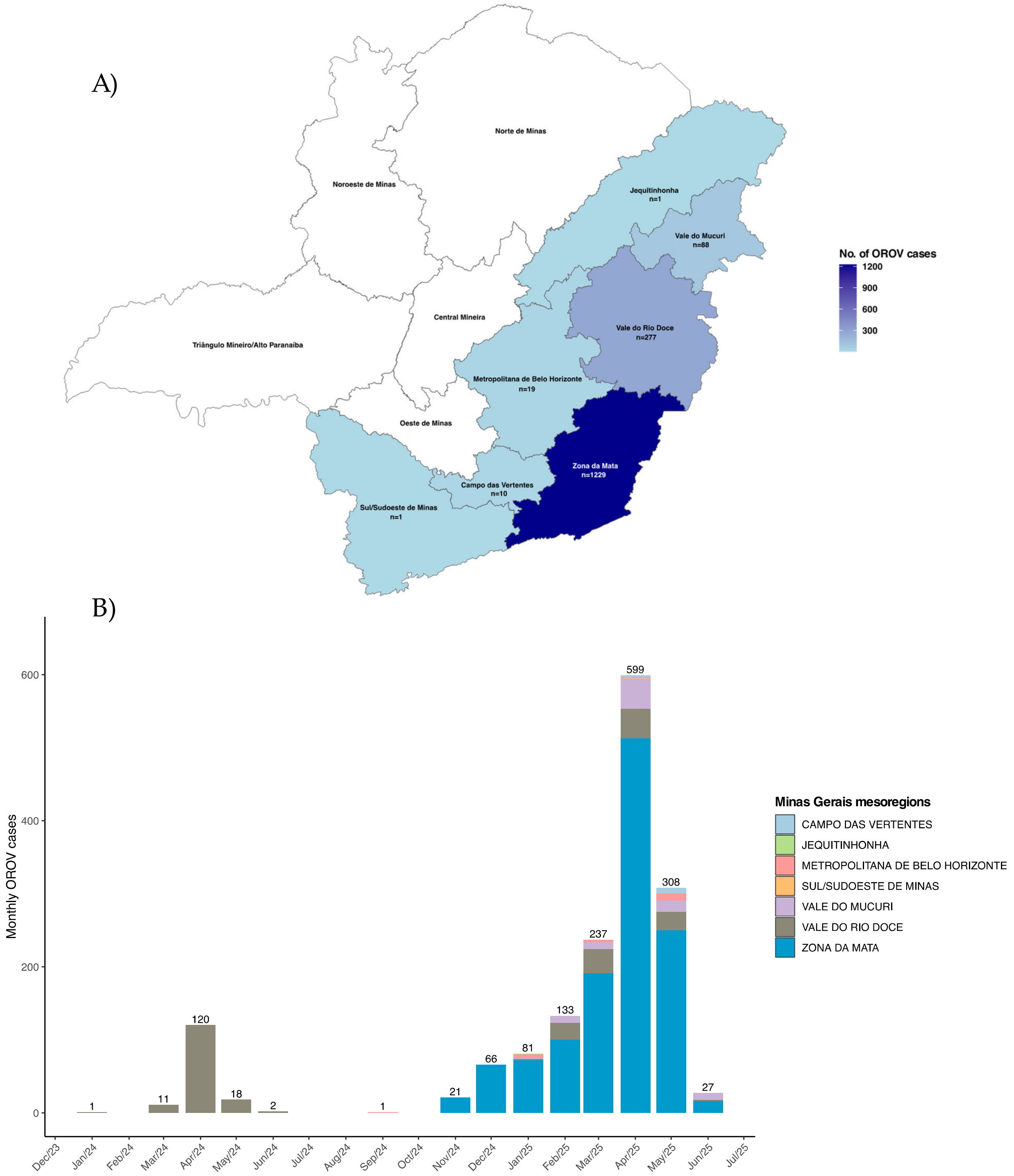

Figure S1. Distribution of *Orthobunyavirus oropouchense* (OROV) cases in Minas Gerais, Brazil. A) Map of the state of Minas Gerais showing the number of OROV cases by mesoregion, reported between 2023 and 2025. B) Monthly distribution of OROV cases confirmed by molecular diagnosis between December 2023 and July 2025, stratified by mesoregion in Minas Gerais.

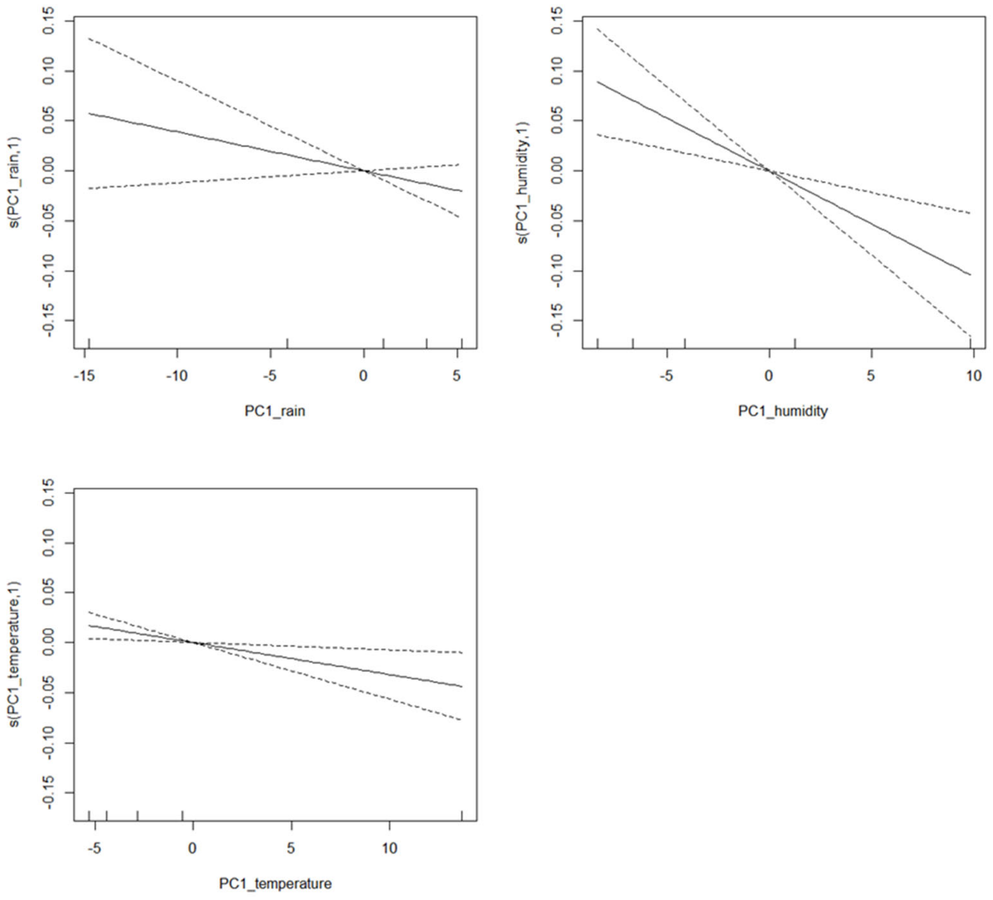

Figure S2. Partial effects of climatic principal components on standardized abundance estimated using a generalized additive mixed model (GAMM). Solid lines represent the estimated smooth effects of (a) precipitation (PC1_rain), (b) air humidity (PC1_humidity), and (c) temperature (PC1_temperature), while dashed lines indicate ±95% confidence intervals. Smooth terms were fitted with thin-plate regression splines (k = 5). The model included random intercepts for communities and species.

Table S1. Geographic coordinates and characteristics of the ecological communities (A–E) sampled in Minas Gerais, Brazil.

Community	Health region	Mesoregion	Latitude	Longitude	Altitude (m)	Landscape type
A	Cataguases	Zona da Mata	21°24'22.6" S	42°41'49.5 "W	184	Peri-urban
B	Cataguases	Zona da Mata	21°17'42.5" S	42°43'38.6 "W	354	Peri-urban
C	Ubá	Zona da Mata	21°09'52.6" S	42°32'27.9 "W	383	Rural settlements
D	Ubá	Zona da Mata	21°09'17.9" S	43°04'25.1 "W	376	Rural settlements
E	Teófilo Otoni	Vale do Mucuri	17°58'44.8" S	041°29.3" W	373	Rural settlements

Table S2. Physiological state of female *Culicoides* captured in five communities within outbreak areas in Minas Gerais, Brazil. Abbreviations: En. = Engorged; Gr. = Gravid; Nu. = Nulliparous; Pa. = Parous; Un. = Undefined.

Community	Physiological state	*C. foxi*	*C. leopoldoi*	*C. limai*	*C. paraensis*	*C. pusillus*	Total
A	En.	0	1	0	0	1	2
	Gr.	0	2	0	2	1	5
	Nu.	0	1	0	0	0	1
	Pa.	0	1	1	5	0	7
	Un.	0	0	0	2	1	3
B	En.	0	3	0	0	0	3
	Gr.	0	14	0	0	1	15
	Nu.	0	5	0	0	0	5
	Pa.	0	9	0	1	0	10
	Un.	0	0	0	0	1	1
C	En.	0	0	0	1	0	1
	Gr.	0	0	0	0	0	0
	Nu.	0	0	0	0	0	0
	Pa.	0	0	0	12	0	12
	Un.	0	0	0	0	0	0
D	En.	0	3	0	0	0	3
	Gr.	0	1	0	0	0	1
	Nu.	0	0	0	0	0	0
	Pa.	0	4	0	2	0	6
	Un.	0	0	0	0	0	0
E	En.	0	22	0	0	0	22
	Gr.	0	84	0	0	0	84
	Nu.	0	41	0	0	0	41
	Pa.	0	56	0	1	0	57
	Un.	1	32	0	0	0	33

Table S3. Results of a Generalized Additive Mixed Model (GAMM) assessing the relationship between standardized *Culicoides* abundance and three principal components summarizing climatic variation: PC1_rain (rainfall metrics), PC1_humidity (air humidity metrics), and PC1_temperature (temperature metrics). For smooth terms (s()), edf = estimated degrees of freedom and Ref.df = reference degrees of freedom. The test statistic is t for the parametric term (Intercept) and F for the smooth terms. Significance was evaluated using the mgcv default thresholds (* $p < 0.05$; ** $p < 0.01$). The model explains 37.2% of the variance in standardized abundance ($R^2 = 0.372$; n = 394; scale estimate = 0.0005) after accounting for the random effects of community and species.

Term	Estimate/edf	Std. Error /Ref.df	t/F	p-value
Intercept	0.02237	0.01105	t = 2.025	0.0435*
s(PC1_rain)	edf = 1	Ref.df = 1	F = 2.253	0.13417
s(PC1_humidity)	edf = 1	Ref.df = 1	F = 10.958	0.00102**
s(PC1_temperature)	edf = 1	Ref.df = 1	F = 6.505	0.01114*

Supplementary Material S1. GAMM output

This supplementary material provides the model specification and full summary statistics for the generalized additive mixed model (GAMM) used to evaluate environmental effects on effort-corrected and standardized *Culicoides* abundance.

Model specification: A GAMM was fitted using the mgcv package in R with Gaussian errors and an identity link, including smooth terms (k = 5) for the first principal component of precipitation (PC1_rain), air humidity (PC1_humidity), and temperature (PC1_temperature), and random intercepts for community and species.

standardized_abundance ~ s(PC1_rain, k=5) + s(PC1_humidity, k=5) + s(PC1_temperature, k=5) + (1 | community) + (1 | species)
